# Association of GTF2I, NFKB1, and TYK2 Regional Polymorphisms With Systemic Sclerosis in a Chinese Han Population

**DOI:** 10.3389/fimmu.2021.640083

**Published:** 2021-06-23

**Authors:** Chenxi Liu, Songxin Yan, Haizhen Chen, Ziyan Wu, Liubing Li, Linlin Cheng, Haolong Li, Yongzhe Li

**Affiliations:** ^1^ Department of Clinical Laboratory, State Key Laboratory of Complex Severe and Rare Diseases, Peking Union Medical College Hospital, Chinese Academy of Medical Science and Peking Union Medical College, Beijing, China; ^2^ Department of Clinical Laboratory, The First Hospital of Jilin University, Jilin, China; ^3^ Department of Rheumatology and Clinical Immunology, Peking Union Medical College Hospital, Key Laboratory of Rheumatology and Clinical Immunology, Ministry of Education, Chinese Academy of Medical Sciences & Peking Union Medical College, Beijing, China

**Keywords:** systemic sclerosis, single nucleotide polymorphism, genetic susceptibility, GTF2I, NFKB1

## Abstract

**Objectives:**

Systemic sclerosis (SSc) is an uncommon autoimmune disease that varies with ethnicity. Single nucleotide polymorphisms (SNPs) in the GTFSI, NFKB1, and TYK2 genes have been reported to be associated with SSc in other populations and in individuals with various autoimmune diseases. This study aimed to investigate the association between these SNPs and susceptibility to SSc in a Chinese Han population.

**Method:**

A case-control study was performed in 343 patients with SSc and 694 ethnically matched healthy controls. SNPs in GTF2I, NFKB1, and TYK2 were genotyped using a Sequenom MassArray iPLEX system. Association analyses were performed using PLINK v1.90 software.

**Result:**

Our study demonstrated that the *GTF2I* rs117026326 T allele and the *GTF2I* rs73366469 C allele were strongly associated with patients with SSc (*P* = 6.97E-10 and *P* = 1.33E-08, respectively). Patients carrying the *GTF2I* rs117026326 TT genotype and the *GTF2I* rs73366469 CC genotype had a strongly increased risk of SSc (*P* = 6.25E-09 and *P* = 1.67E-08, respectively), and those carrying the *NFKB1* rs1599961 AA genotype had a suggestively significantly increased risk of SSc (*P* = 0.014). Moreover, rs117026326 and rs73366469 were associated with SSc in different genetic models (additive model, dominant model, and recessive model) (*P* < 0.05) whereas rs1599961 was associated with SSc in the dominant genetic model but not in the addictive and recessive models (*P* = 0.0026). *TYK2* rs2304256 was not significantly associated with SSc in this study.

**Conclusion:**

*GTF2I* rs117026326 and rs73366469 SNPs were strongly associated with SSc in this Chinese Han population. *NFKB1* rs1599961 showed a suggestive association with SSc, and no significant association was found between *TYK2* rs2304256 and SSc in this Chinese Han population.

## Introduction

Systemic sclerosis (SSc) is an autoimmune disease characterized by limited or diffuse skin thickening, tissue fibrosis, and the immune response. Most patients with SSc develop skin thickening with variable organ involvement, including interstitial lung disease (ILD), pulmonary arterial hypertension (PAH), and renal crisis (1). The etiology and pathogenesis of SSc involves genetic, epigenetic, and environmental factors. Genetic factors in particular play an important role in susceptibility to the disease (2).

At present, there have been many genetic studies on SSc, including genome-wide association studies (GWAS) and Immunochip analyses (3–5). Several immune loci related to SSc susceptibility have been identified. Although great progress has been made in the past few years, our understanding of the genetic background of SSc remains limited, and the number of convincing SSc genetic markers only accounts for a small part of the total genetic variance. Therefore, further genetic research is of the utmost importance to better understand the pathogenic process of SSc, such as performing an association analysis of candidate single nucleotide polymorphisms (SNPs).

GTF2I is a gene located on the long arm of chromosome 7 (7q11.23) and encodes for a signal-induced transcription factor that functions as a universal regulator of numerous cellular processes. Many genetic studies have found that GTF2I is associated with multiple autoimmune rheumatic diseases. A study from six East Asian cohorts identified SNPs located at the GTF2I region that were associated with systemic lupus erythematosus (SLE) susceptibility (6). A study on rheumatoid arthritis (RA) in Asia confirmed that the GTF2I region may be a pathogenic variant and identified the largest-ever effect by heterogeneity mapping (7). A recent study has reported that this region was associated with susceptibility to SLE and SSc in a Japanese population (7).

Conversely, the deregulation of nuclear factor κB (NF-κB) can lead to multiple autoimmune disorders, including type 1 diabetes (T1D), SLE, and RA (8). NF-κB is a ubiquitous transcription factor of the Rel proto-oncogene family. It regulates the expression of several genes involved in inflammation and immune response. The NF-κB family is composed of five related proteins, including p50 or NF-κB1. The NF-κB signaling pathway plays an important role in the development and progress of RA both in vitro and in vivo (9). A study found that NF-κB1-deficient mice developed more fibrosis compared with wild-type mice (10). Moreover, NFKB1 loci have shown a suggestive association with SSc in a Middle Eastern population (11).

The Janus kinase family (JAK1, 2, and 3 and TYK2) has been recognized as important regulators of inflammation and immune processes. The kinase encoded by TYK2 can regulate the signal transduction of a variety of pro-inflammatory cytokines, including IL12, IL23, and type 1 interferon (IFNα). A recent fine mapping genetic study of RA found that three TYK2 protein coding variants are the most likely pathogenic variants that lead to the associated signals in this region (12). Simultaneously, functional prediction tools confirmed that the TYK2 variant can also cause damage such as SLE and inflammatory bowel disease. Furthermore, the TYK2 variant was reported to be associated with SSc in the European population (4).

Considering the genetic overlap of autoimmune diseases and the association of these genes with SSc in other populations, we hypothesized that some of the related tag SNPs of GTF2I, NFKB1, and TYK2 may also contribute to susceptibility to SSc in the Chinese Han population. Therefore, this study aimed to determine whether SNPs in GTF2I, NFKB1, and TYK2 were associated with SSc in a Chinese Han population and to explore the correlation of these loci and SSc clinical characteristics.

## Methods

### Study Populations

The SSc cohort employed in this study included 342 clinically diagnosed patients with SSc who fulfilled the 2013 ACR/EULAR classification criteria for SSc (13). Patients were classified with diffuse cutaneous systemic sclerosis (dcSSc) and limited cutaneous systemic sclerosis (lcSSc), according to the LeRoy criteria (14). Tests for the two SSc-related specific autoantibodies anti-topoisomerase I (ATA) and anti-centromere (ACA), which are most commonly tested in clinical applications, were performed in accordance with standard clinical laboratory methods. In total, 694 healthy subjects admitted to the Peking Union Medical College Hospital Health Examination Center for physical examination were recruited as negative controls for this study. ILD was identified with high-resolution computed tomography, and PAH was identified with right heart catheterization (PAH was diagnosed as a mean pulmonary pressure of ≥25 mmHg). All serum samples were stored at −80°C until use. All samples were obtained from patients recruited from the Peking Union Medical College Hospital (PUMCH). This study was approved by the Ethics Committee of the PUMCH, and all recruited participants provided informed consent.

### DNA Extraction and Genotyping

The genomic DNA of each patient was extracted from peripheral blood samples using Tiangen DNA kits (Tiangen, Beijing, China). MassArray Assay Design 4.0 software (Sequenom, San Diego, CA, USA) was used to design the primers for the multiplex polymerase chain reaction (PCR) and locus-specific extension. Approximately 10–20 ng of DNA was amplified by multiplex PCR, and the DNA was genotyped using a Sequenom MassArray system (San Diego, CA) according to the manufacturer’s instructions. The final product was desalted and added to a 384-element Spectro CHIP array (Sequenom). Allele detection was performed by matrix-assisted laser desorption ionization-time-of-flight mass spectrometry (MALDI-TOF MS). Mass spectrometry data and genotype were analyzed using MassArray Typer 4.0 software.

### Statistical Analysis

PLINK v1.90, SPSS Statistics v.25 (IBM), and Prism v.8 (GraphPad) were used to analyze the data. The Chi-square test was applied to analyze Hardy–Weinberg equilibrium (HWE). If the *P* value of the SNP in the healthy control group was less than 0.05, the control group deviated from HWE and would be excluded. The associations between alleles and SSc were assessed with a logistic regression analysis, and the associations between genotypes and SSc were assessed with a Chi-square test. Three logistic regression models (additive, dominant, and recessive) and subgroup stratification analyses were assessed with a logistic regression analysis. All regressions were subjected to age adjustment. The odds ratio (OR) and confidence interval (95% CI) were calculated, and *P* values were statistically significant when <0.05 and were bolded in **Tables 3**–**5**. Finally, haplotype analysis and linkage disequilibrium were conducted using Haploview software v4.2.

## Results

### Clinical Characteristics of Patients With SSc and Healthy Controls

Our study included 342 patients with SSc [mean age ± standard deviation (SD): 48.1 ± 12.5 years; 34 males and 308 females] and 694 healthy controls (mean age ± SD: 49.4 ± 11.0 years; 60 males and 634 females) (**Table 1**). Among the 342 patients with SSc, 21 had dcSSc and 153 had lcSSc (168 patients with SSc had no classification result). In total, 219 (64.0%) patients with SSc had ILD and 74 (21.6%) had PAH.

**Table 1:** Clinical characteristics of patients with SSc and healthy controls.

	SSc Patients	Healthy Controls
Number	342	694
Mean age ± SD	48.1 ± 12.5	49.4 ± 11.0
Gender, male/female	34 /308	60/634
dcSSc, n (%)	21 (6.1%)	–
lcSSc, n (%)	153 (44.7%)	–
ILD, n (%)	219 (64.0%)	–
PAH, n (%)	74 (21.6%)	–

SSc, systemic sclerosis; ILD, interstitial lung disease; PAH, pulmonary arterial hypertension; dcSSc, diffuse cutaneous systemic sclerosis; lcSSc, limited cutaneous systemic sclerosis.

### Allele and Genotype Frequencies in SSc Patients and Healthy Controls

**Figure 1:**
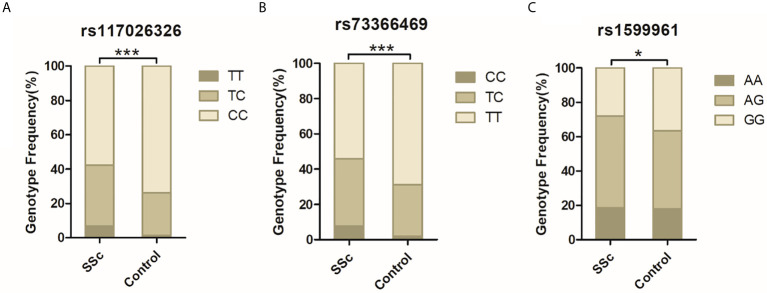
The genotype distributions of the three significant SNPs [**(A)** rs117026326, **(B)** rs73366469, and **(C)** rs1599961] in the patients with SSc and healthy controls are summarized. ***P < 0.001. *P < 0.05.

DNA from all subjects in the study was genotyped for the four selected SNPs, and the findings are summarized in **Table 2**. Four SNPs in the control group fulfilled the HWE criteria (*P* > 0.05). The allele and genotype frequencies in the patients with SSc and healthy controls are presented in **Table 3**. The rs117026326 and rs73366469 loci of the *GTF2I* gene were strongly associated with SSc. The T allele frequency of rs117026326 was higher in patients with SSc than in controls (24.6% vs. 13.9%, OR: 2.22, 95% CI: 1.72–2.86, *P* = 6.97E-10). The C allele frequency of rs73366469 was higher in patients with SSc than in controls (26.8% vs. 16.6%, OR: 2.02, 95% CI: 1.58–2.57, *P* = 1.33E-08). Furthermore, the genotype frequencies differed between patients with SSc and healthy controls. The TT genotype of rs117026326 had a significantly increased risk of SSc compared with the TC and CC genotypes (*P* = 6.25E-09). The CC genotype of rs73366469 had a significantly increased risk of SSc compared with the TC and TT genotypes between patients with SSc and healthy controls (*P* = 1.67E-08). Additionally, despite the lack of significant differences in the allele frequency in rs1599961 of the *NFKB1* gene, the AA genotype had a suggestively significantly increased risk of SSc compared with the AG and GG genotypes between patients with SSc and healthy controls (*P* = 0.014). The genotype distributions of the three significant SNPs ((A) rs117026326, (B) rs73366469, and (C) rs1599961) in the patients with SSc and healthy controls are summarized in **Figure 1**. However, we did not detect any significant differences in the allele and genotype frequency distributions of rs2304256 of the *TYK2* gene between patients with SSc and healthy controls (*P* > 0.05).

**Table 2:** Characteristics of four SNPs.

Gene	SNP	Chromosome	Position	Distance to TSS^**^	Function	Allele	MAF in CHB
*GTF2I*	rs117026326	7	74711703	54,040	intron variant	C>T	0.117
*GTF2I*	rs73366469^*^	7	74619286	38,394	regulatory region variant	T>C	0.155
*NFKB1*	rs1599961	4	102522412	21,083	intron variant	G>A	0.388
*TYK2*	rs2304256	19	10364976	15,700	missense variant	C>A	0.456

^*^The distance between rs73366469 and GTF2IRD1 is 1 kb, whereas the distance between rs73366469 and GTF2I is 28 kb. Here, we assign rs73366469 to GTF2I. ^**^TSS, transcription start sites.

**Table 3:** Allele and genotype frequencies in patients with SSc and controls.

Gene	SNP	Allele Frequency (%)				Genotype Frequency (%)			
		Allele	Case/control (%)	*P*	OR (95%CI)	Genotype	Case/control (%)	*P*	χ^2^
*GTF2I*	rs117026326	T	166/192 (24.6/13.9)	**6.97E-10**	2.22 (1.72-2.86)	TT	23/10 (6.8/1.4)	**6.25E-09**	37.78
		C	510/1190 (75.4/86.1)			TC	120/172 (35.5/24.9)		
						CC	195/509 (57.7/73.7)		
	rs73366469	C	181/228 (26.8/16.6)	**1.33E-08**	2.02 (1.58-2.57)	CC	26/14 (7.7/2.0)	**1.67E-08**	35.82
		T	495/1148 (73.2/83.4)			TC	129/200 (38.2/29.1)		
						TT	183/474 (54.1/68.9)		
*NFKB1*	rs1599961	A	308/560 (40.8/45.3)	0.086	1.19 (0.97-1.46)	AA	63/124 (18.5/18.1)	**0.014**	8.51
		G	372/814 (59.2/54.7)			AG	182/312 (53.5/45.4)		
						GG	95/251 (28.0/36.5)		
*TYK2*	rs2304256	A	296/626 (45.4/43.5)	0.592	0.95 (0.77-1.56)	AA	67/140 (19.7/20.3)	0.599	1.03
		C	384/754 (54.6/56.5)			CA	162/346 (47.7/50.1)		
						CC	111/204 (32.6/29.6)		

SSc, systemic sclerosis.

### Genetic Models Determined by Logistic Regression Analysis

Three genetic models were analyzed to explore the effect of different genotypes on SSc (**Table 4**). rs117026326 was significantly associated with SSc in the genetic addictive, dominant, and recessive models (*P* = 1.62E-09, 1.23E-07, and 9.72E-06, respectively). rs73366469 was significantly associated with SSc in the genetic addictive, dominant, and recessive models (*P* = 6.93E-09, 3.26E-06, and 1.91E-06, respectively). rs1599961 was significantly associated with SSc in the genetic dominant model (*P* = 0.0026) but not in the genetic addictive or recessive models (*P* > 0.05), which indicates that the AA and AG genotypes had a significantly increased risk of SSc compared with the GG genotype between patients with SSc and healthy controls. However, rs2304256 was not associated with SSc in any of the three genetic models (*P* > 0.05).

**Table 4:** Genetic models determined by logistic regression analysis.

Gene	SNP	Addictive Model		Dominant Model		Recessive Model	
		*P*	OR (95%CI)	*P*	OR (95%CI)	*P*	OR (95%CI)
*GTF2I*	rs117026326	**1.62E-09**	2.23 (1.72-2.89)	**1.23E-07**	2.25 (1.67-3.05)	**9.72E-06**	6.24 (2.77-14.05)
	rs73366469	**6.93E-09**	2.11 (1.64-2.71)	**3.26E-06**	2.01 (1.50-2.69)	**1.91E-06**	5.76 (2.80-11.85)
*NFKB1*	rs1599961	0.089	1.19 (0.97-1.46)	**0.0026**	1.61 (1.18-2.20)	0.526	0.89 (0.61-1.29)
*TYK2*	rs2304256	0.591	0.95 (0.78-1.16)	0.292	0.85 (0.62-1.15)	0.786	1.05 7(0.74-1.50)

The above data revealed that rs117026326 might be consistent with rs73366469. A linkage disequilibrium analysis was performed to evaluate this potential, and the results indicated that the two SNPs were in median linkage disequilibrium (D’ = 0.942, r^2^ = 0.756).

### Associations Between SNPs and Patients With SSc With Different Clinical Characteristics

To further investigate the association between these SNPs and SSc susceptibility, we performed subgroup analyses (**Table 5**). We compared the frequencies of SNPs in patients with SSc with ILD with those of patients with SSc without ILD and with those of healthy controls. We performed the same subgroup analyses for PAH. We also compared the frequencies of SNPs in patients with dcSSc patients with those in patients with lcSSc as well as those in autoantibody-positive patients with those in autoantibody-negative patients. According to our results, SSc–ILD was associated with *GTF2I* rs117026326 (*P* = 1.34E-04) and *GTF2I* rs73366469 (*P* = 2.22E-04). SSc-non-ILD was associated with *GTF2I* rs117026326 (*P* = 5.74E-06), *GTF2I* rs73366469 (*P* = 2.58E-05), and *NFKB1* rs1599961 (*P* = 0.011). SSc–PAH was associated with *GTF2I* rs117026326 (*P* = 3.54E-04) and *GTF2I* rs73366469 (*P* = 3.10E-04). SSc–non-PAH was associated with *GTF2I* rs117026326 (*P* = 1.46E-05) and *GTF2I* rs73366469 (*P* = 7.37E-05). No statistically significant association was found between *TYK2* rs2304256 and any SSc clinical characteristics (*P* > 0.05). No statistically significant association was found between the four SNPs and the two autoantibodies (*P* > 0.05).

**Table 5:** Associations between SNPs and patients with SSc with different clinical characteristics.

Clinical characteristics	rs117026326 (*GTF2I*)		rs73366469 (*GTF2I*)		rs1599961 (*NFKB1*)		rs2304256 (*TYK2*)	
	*P*	OR	*P*	OR	*P*	OR	*P*	OR
SSc–ILD vs SSc–non-ILD	0.129	0.74 (0.50–1.09)	0.179	0.77 (0.52–1.13)	0.083	0.72 (0.50–1.04)	0.426	1.15 (0.81–1.63)
SSc–ILD vs HC	1.34E–04^*^	0.58 (0.44–0.77)	2.22E–04^*^	1.66 (1.27–2.17)	0.307	1.12 (0.90–1.38)	0.97	1.00 (0.81–1.25)
SSc–non-ILD vs HC	5.74E–06^*^	2.38 (1.64–3.46)	2.58E–05^*^	2.18 (1.52–3.14)	0.011^*^	1.49 (1.09–2.02)	0.388	0.87 (0.64–1.19)
SSc–PAH vs SSc–non-PAH	0.99	1.0 (0.63–1.60)	0.78	1.07 (0.67–1.70)	0.796	1.06 (0.69–1.62)	0.735	1.07 (0.71–1.61)
SSc–PAH vs HC	3.54E–04^*^	2.13 (1.41–3.23)	3.10E–04^*^	2.09 (1.40–3.12)	0.081	1.34 (0.96–1.87)	0.844	1.04 (0.74–1.46)
SSc–non-PAH vs HC	1.46E–05^*^	2.12 (1.51–2.99)	7.37E–05^*^	1.96 (1.40–2.73)	0.074	1.28 (0.98–1.67)	0.788	0.96 (0.73–1.27)
dcSSc vs lcSSc	0.601	1.23 (0.57–2.68)	0.68	1.17 (0.55–2.50)	0.448	0.77 (0.39–1.51)	0.134	0.61 (0.32–1.16)
ATA (+) vs ATA (–)	0.088	0.66 (0.40–1.07)	0.26	0.77 (048–1.22)	0.72	1.09 (0.69–1.70)	0.67	1.10 (0.72–1.67)
ACA (+) vs ACA (–)	0.55	0.83 (0.46–1.51)	0.86	0.95 (0.54–1.69)	0.77	0.92 (0.52–1.62)	0.51	0.84 (0.51–1.40)

SSc, systemic sclerosis; HC, healthy controls; ILD, interstitial lung disease; PAH, pulmonary arterial hypertension; ATA, anti-topoisomerase I antibody; ACA, anti-centromere antibody. ^*^The P value was still < 0.05 after Bonferroni correction.

### Functional Annotations of SNPs

We annotated the epigenetic regulatory features for the fours SNPs using Haploreg (15) and GWAS4D (16) online tools. **Supplementary Table 1** shows the results of the functional annotations for binding motifs and epigenetic marks by Haploreg. GWAS4D incorporates 127 tissue/cell type-specific epigenomic data, integrates and refines transcription factor (TF) motifs from eight public resources, uniformly processes Hi-C data, etc. The results of annotation with GWAS4D are shown in **Supplementary Tables 2**–**7**.

## Discussion

To our knowledge, this is a candidate gene association analysis that describes the association between functional *GTF2I*, *NFKB1*, and *TYK2* polymorphisms and SSc in a Chinese Han population. This study demonstrated that the *GTF2I* polymorphisms were strongly associated with SSc in the Chinese Han population and that the NFKB1 polymorphism was suggestively associated with SSc in the Chinese Han population.

*GTF2I* encodes general transcription factor Iii (TFII-I). It binds to the initiator element (Inr) and E-box element in promoters and functions as a regulator of transcription (17). TFII-I is involved in the regulation of transcription, signal transduction, and immune cell signaling (18). Among its related pathways are RNA polymerase II transcription initiation and promoter clearance and the B cell receptor signaling pathway (KEGG). The *GTF2I* gene can regulate immunoglobulin heavy chain transcription in B cells (19). Makeyev et al. confirmed that the impaired expression of *GTF2I* could contribute to the etiology of Williams syndrome (20). Novel studies have shown that *GTF2I* is involved in an array of human diseases including neurocognitive disorders and cancer (21, 22). Our previous GWAS showed that *GTF2I* was the most strongly associated gene in Chinese Han patients with primary Sjögren’s syndrome (pSS) (23). Several association studies of candidate genes have revealed that the *GTF2I* gene is an important genetic susceptibility factor in SLE and RA (7, 24). Furthermore, SSc, pSS, SLE, and RA are all connective tissue diseases and share many clinical features, particularly in aspects of immune activation (25). These diseases usually coexist in patients in the form of overlap syndrome. Therefore, the *GTF2I* gene may also play an essential role in SSc. Our results indeed confirmed that *GTF2I* SNPs rs117026326 and rs73366469 were strongly associated with SSc in the Chinese Han population. Interestingly, rs73366469 is located within conserved enhancers and overlaps with transcription start sites (TSS) for *GTF2I*, thereby demonstrating the meaningful regulation of *GTF2I* (24). Additionally, rs117026326 and rs73366469 displayed consistent allele, genotype, genetic model, and clinical association results because of their medium linkage disequilibrium. Therefore, the two SNPs were most likely to have regulatory functions, which can also be verified by functional annotation using the online tools.

We also compared SSc accompanied with ILD and SSc accompanied without ILD and found no association between the two factors. And there was no previous study assessing the association between *GTF2I* and ILD. The significant result identified when the two groups were compared with healthy controls was possibly a result of the role of SSc disease itself instead of the role of ILD or non-ILD. The same principle is also applicable to the interpretation of the PAH results.

*NFKB1* encodes a 105 kD protein that can undergo cotranslational processing by the 26S proteasome to produce a 50 kD protein. The 50-kD protein is a DNA binding subunit of the NF-kappa-B (NF-κB) protein complex. NF-κB is a transcription regulator that is activated by various intra- and extracellular stimuli such as cytokines, oxidant-free radicals, and bacterial or viral products. The inappropriate activation of NF-κB has been associated with a number of inflammatory diseases whereas persistent NF-κB inhibition leads to inappropriate immune cell development. Increased CD8+ T cell apoptosis in SSc was reported to be associated with low levels of NF-κB (26). A meta-analysis suggested a possible association between NFKB1 polymorphisms and certain autoimmune and inflammatory diseases in the Asian population but not in the Caucasian population (27). A GWAS of SSc in Iranian and Turkish populations identified a suggestive association between NFKB1 loci and SSc (11). However, NFKB1 polymorphisms showed no association with RA and ankylosing spondylitis (AS) (28, 29) or with SSc in Brazil (30), which may be due to the genetic differences of the populations. Then, we used the Haploreg online tool to annotated NFKB1 rs1599961. We found that high-LD SNPs rs1585213(r^2^ ≥ 1), rs3774933(r^2^ = 0.99), and rs4647980(r^2^ = 0.84) have multiple protein binding confirmations. It is speculated that these SNPs may affect the transcription factors such as ZNF263, CEBPB, FOXA1 and POL24H8, and then affect the transcription of downstream genes.

In the present study, people carrying the *NFKB1* rs1599961 AA genotype had a suggestively significantly increased risk of SSc in the Chinese Han population; moreover, rs1599961 was related to SSc in the dominant genetic model but not in the addictive or recessive models. This result may have occurred because the frequency of the GG genotype was lower in patients with SSc than in healthy controls. The distribution of rs1599961 significantly differed when comparing patients with SSc without ILD with healthy controls. Thus, *NFKB1* rs1599961 could be a candidate locus involved in SSc, particularly in Chinese Han patients without ILD.

*TYK2* encodes a tyrosine kinase from the Janus kinase family. This protein can bind to the intracellular domain of Th1 and Th2 cytokine receptors and conduct signal transduction through the phosphorylation of receptor subunits. Previous studies have found that *TYK2* polymorphisms are associated with a variety of autoimmune diseases such as RA, SLE, T1D, and multiple sclerosis (31–34). An Immunochip study of SSc found a suggestive association in the *TYK2* region (35). Furthermore, rs2304256 was revealed as a nonsynonymous variant (C>A) located in *TYK2* exon 8, causing the substitution of valine with phenylalanine (c.1084 G>T, Val362Phe) (36). This substitution thus affected the processing of pre-mRNA at exon 8. In addition, the eQTL results showed that rs2304256 enhanced *TYK2* expression in blood. In summary, *TYK2* rs2304256 is not neutral but has a potential impact on autoimmune diseases. *TYK2* rs2304256 was reported to be strongly associated with SSc in a European population (4). However, this significant result was not replicated in the Chinese Han SSc population. No significant association was found between *TYK2* rs2304256 and SSc in the current Chinese Han population in any of the analyses, which may be the result of racial differences.

In conclusion, *GTF2I* rs117026326 and rs73366469 were strongly associated with SSc in this Chinese Han population. *NFKB1* rs1599961 showed a suggestive association with SSc whereas no significant association was found between *TYK2* rs2304256 and SSc in this Chinese Han population.

## Data Availability Statement

The original contributions presented in the study are included in the article/**Supplementary Material**. Further inquiries can be directed to the corresponding author.

## Ethics Statement

The studies involving human participants were reviewed and approved by the Ethics Committee of the PUMCH. The patients/participants provided their written informed consent to participate in this study. Written informed consent was obtained from the individual(s) for the publication of any potentially identifiable images or data included in this article.

## Author Contributions

CL wrote this article. SY and HC conducted laboratory tests. ZW provided assistance with clinical information. LL and LC conducted data collection and data analysis. HL revised this article. YL designed experiments and guidance. All authors contributed to the article and approved the submitted version.

## Funding

This research was supported by the grants from The National Key Research and Development Program of China (2018YFE0207300), the National Natural Science Foundation of China (Grants 81671618 and 81871302) and the CAMS Innovation Fund for Medical Sciences (CIFMS 2017-I2M-3-001and CIFMS 017-I2M-B&R-01). This work was supported by Beijing Key Clinical Specialty for Laboratory Medicine - Excellent Project (No. ZK201000).

## Conflict of Interest

The authors declare that the research was conducted in the absence of any commercial or financial relationships that could be construed as a potential conflict of interest.
